# Neuroendoscopic Approach of a Massive Bilateral Chronic Subdural Hematoma in a Child Using a Single Burr Hole

**DOI:** 10.7759/cureus.12755

**Published:** 2021-01-17

**Authors:** Leopoldo Mandic Ferreira Furtado, José Aloysio Da Costa Val Filho, Camila Moura de Sousa, François Dantas, Júlia da Silva Costa

**Affiliations:** 1 Pediatric Neurosurgery, Vila Da Serra Hospital, Nova Lima, BRA; 2 Neurosurgery, Vila da Serra Hospital, Nova Lima, BRA; 3 Neurological Surgery, Federal University of Vales do Jequitinhonha e Mucuri, Diamantina, BRA; 4 Neurological Surgery, Biocor Instituto, Belo Horizonte, BRA; 5 Neurological Surgery, Hospital Vila da Serra, Belo Horizonte, BRA; 6 Medicine, José Rosário Vellano University, Belo Horizonte, BRA

**Keywords:** neuroendoscopy, chronic subdural hematoma, pediatric head trauma, accidental head trauma, brain injury, minimally invasive, massive hematoma

## Abstract

There are several treatment modalities for the management of subdural fluid collection in infants, such as fontanelle puncture and drainage, burr hole irrigation, and subduroperitoneal shunt. This report describes the case of a girl born with congenital neurological impairment due to severe injury of the brain with unknown etiology. At five months of age, she suffered from head trauma and developed somnolence after three days and was diagnosed with a bilateral massive chronic subdural hematoma. Normal fundoscopy did not confirm the non-accidental head trauma. Neuroendoscopy using a single burr hole was performed and complete drainage was achieved. Arachnoid tearing was observed during the procedure. Postoperatively, the patient showed clinical improvement, and brain expansion was observed after one month. The main advantages of neuroendoscopy for bilateral massive chronic subdural hematoma are accurate visualization of the space, minimal invasiveness, and treatment of both sides with reliable drainage control.

## Introduction

The management of subdural fluid collection in infants is controversial, and several treatment modalities have been reported, such as fontanelle puncture and drainage, burr hole irrigation, and subduroperitoneal shunt [1]. A few studies have also reported neuroendoscopy for evaluating chronic subdural hematomas [2-4]. The main advantages of neuroendoscopy are accurate visualization of the subdural space and minimal invasiveness [5].

This report describe the case of an unusual massive bilateral chronic hematoma in a five-month-old girl that was successfully treated using neuroendoscopy via a single burr hole.

## Case presentation

**Figure 1:**
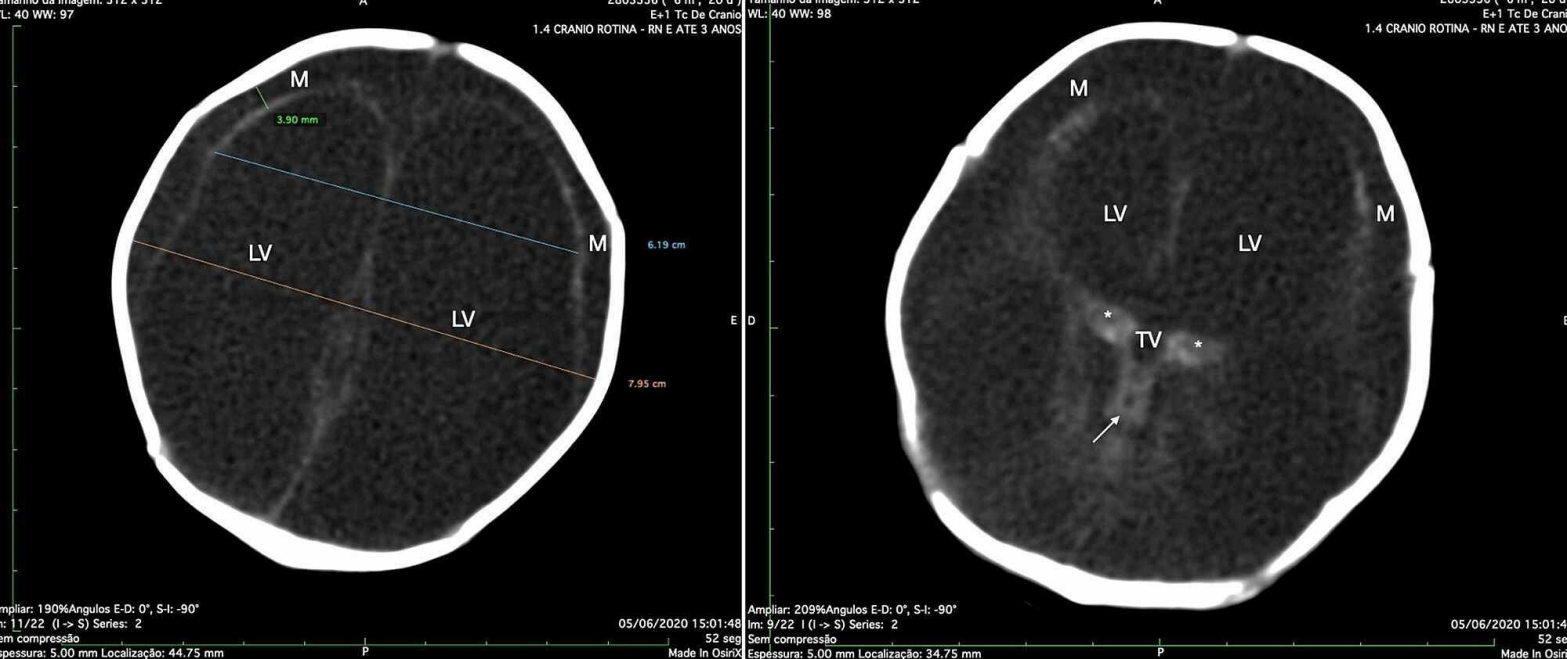
Brain image of a child with congenital brain injury before the head trauma CT scan of the brain without contrast of the patient at two months of age, showing a diffuse severe injury of the brain, presented as a hypodense parenchyma and thin cortical mantle (M) and enlarged compensatory LVs (Evans’ index = 0.77) (left). Thalamus (*), TV, and midbrain (white arrow) are identified (right). LV, lateral ventricle; TV, third ventricle.

A five-month-old girl was brought to the emergency room with a history of falling in the shower one week before and development of somnolence three days later. Previous history examination showed that the patient was born with a diffuse brain lesion of no defined etiology, and computed tomography (CT) of the brain at two months showed a compromised brain and compensatory enlargement of lateral ventricles associated with a thin cortical mantle (Figure 1).

However, the patient grew with no head measurement abnormalities, and the fontanelles present were small.

At admission, after the fall and head trauma, she presented with somnolence, a Glasgow Coma Scale score of 10, sustained bradycardia (70 bpm) without hypertension, and hypothermia (33°C). The oxygen saturation was 98% at ambient air supply, and no bruises were noted in the physical examination.

**Figure 2:**
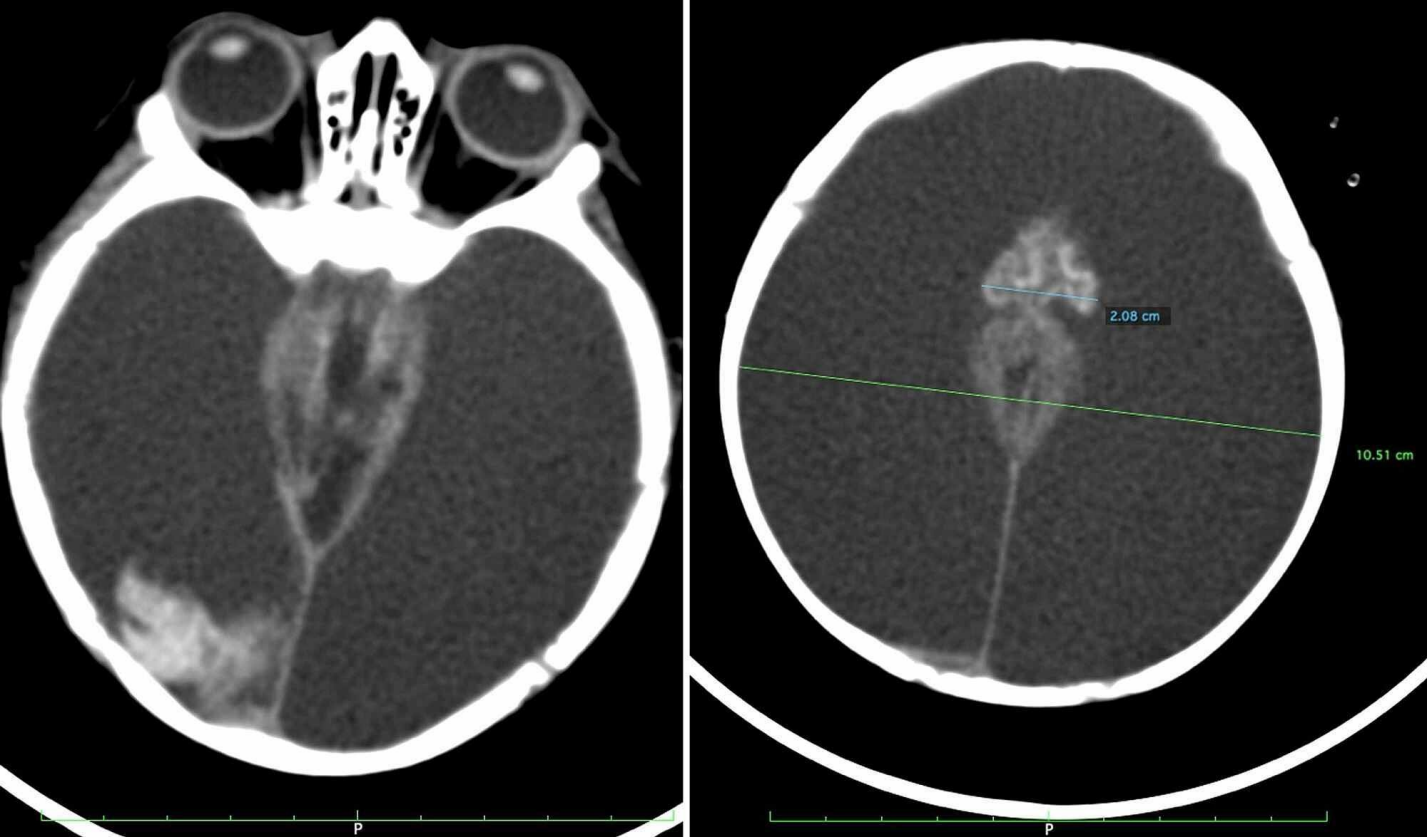
Massive subdural collection on CT scan one week after head trauma CT scan of the brain without contrast showing massive hypointense extracerebral fluid collection associated with hyperintense imaging, occupying the occipital region on the right side of the intracranial compartment (left). Brain collapse can seen at the midline (right).

**Figure 3:**
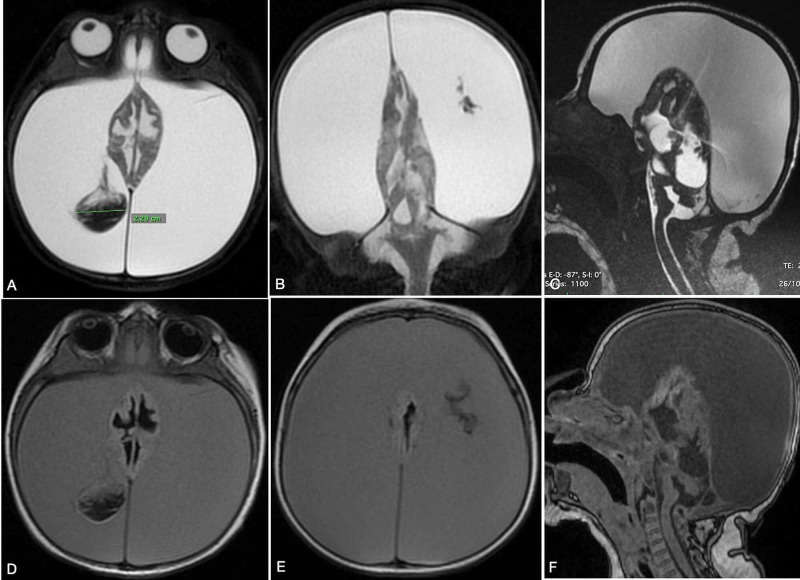
Massive subdural collection on MRI Axial T2-weighted MRI showed a peduncular hypointense mass in the collapsed brain and hyperintense subdural fluid collection (A). Coronal T2-weighted MRI showing a midline collapsed brain (B). Sagittal T2-weighted MRI showing compression of the posterior fossa content by a supratentorial increased hypertensive compartment (C). T1-weighted MRI displaying hypo-/isointense subdural fluid collection (D). Axial T1-weighted MRI high-level slice showing a massive subdural hypo-/isointense mass collapsing the brain (E). Sagittal T1-weighted MRI showed compression of the posterior fossa.

The patient underwent CT and MRI of the brain, which showed a massive bilateral subdural hematoma with two hyperdense masses adherent to the collapsed brain (Figures 2-3).

The non-accidental head trauma was ruled out after psychological assessment and the following measures. An ophthalmologist performed fundoscopy and observed not retinal abnormalities such as hemorrhage. In addition, the patient showed good nutrition conditions, and radiographs of her long bones showed no fractures, even in other periods. 

Because of the massive fluid collection in the subdural space, we decided to perform neurosurgical intervention using neuroendoscopy, expecting to have better navigation in this case. Under general anesthesia, the patient was positioned in the supine position, with the head in a neutral position in a horseshoe headrest. A unique burr hole was made in the frontal region (Video 1).

**Video 1:** Neuroendoscopic technique Introducing the neuroendoscope into the right lateral intracranial subdural compartment shows hemorrhage and turbid liquid. The collapsed brain is observed after clearance, in addition to an arachnoid tearing. Contralateral drainage was undertaken after enlargement of the natural overture of the falx cerebri. At the end of the procedure, the inner surface of the dura is observed in the middle and anterior fossa.

After durotomy, we drained the hypertensive and turbid fluid. Next, we introduced a peel-away sheet into the subdural space and inserted a rigid neuroendoscope with a zero-degree optic. After irrigation of the right subdural space, the hematoma was identified adherent to the arachnoid surface, which also presented a laceration. The falx cerebri presented with natural holes. After enlargement, the contralateral hemorrhage was drained. At the end of the procedure, we observed the entire middle fossa and the anterior fossa with ethmoidal arteries (Video). No drains were introduced. There was an immediate improvement of the heart rate and the level of consciousness. The patient received the discharge from the hospital after one week after the procedure. No complications were observed.

**Figure 4:**
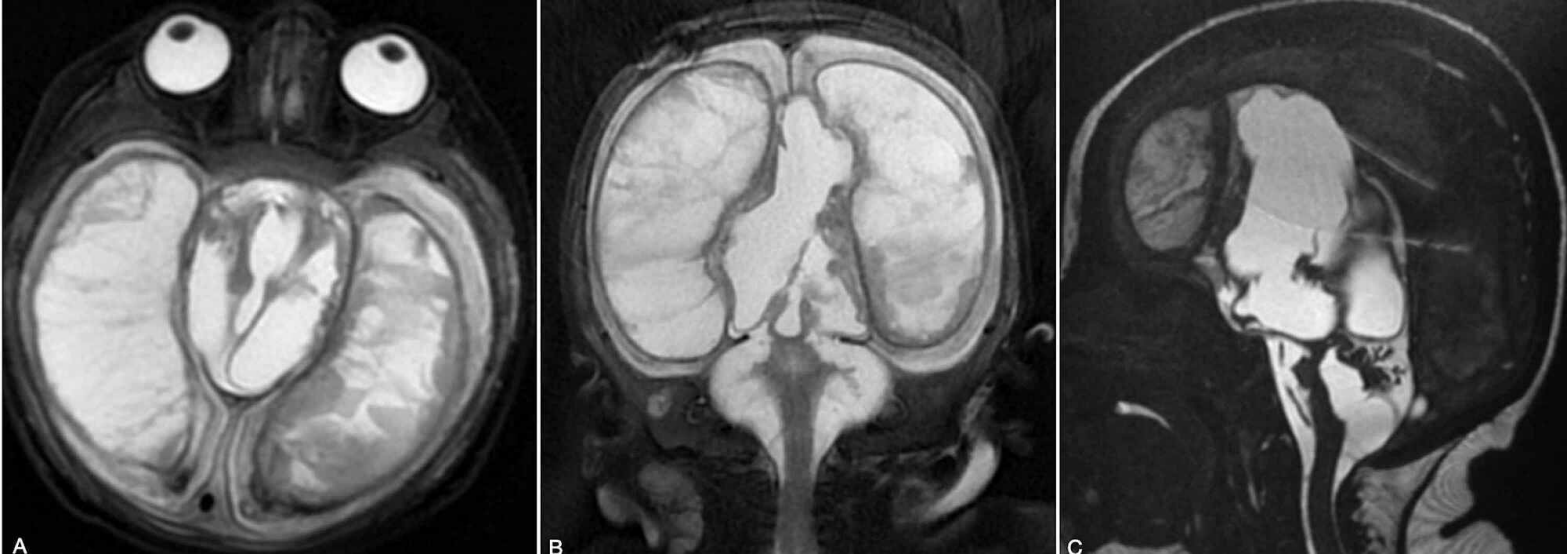
Dysmorphic brain expansion MRI after one month of endoscopy. Axial T2-weighted MRI showing brain expansion (A). Trabeculated imaging inside the brain (B). Improvement of compression of the posterior fossa (C).

MRI obtained 30 days after the procedure showed brain expansion with a thin cortical mantle (Figure 4).

.

## Discussion

Neuroendoscopy is commonly indicated for the treatment of intracranial diseases, such as hydrocephalus, intraventricular cysts, arachnoid cysts, and a trapped fourth ventricle, due to the reliability and accuracy of a neuroendoscope to appreciate small details with a nitid image and improve the visualization of deep structures [6, 7]. However, not many studies have reported the application of neuroendoscopy to treat pathologies on the surface of the brain, such as subdural hematoma. The endoscope could play a role in large subdural fluid collections, similar to its role in intraventricular diseases. The main advantage of neuroendoscopy to treat surface diseases such as subdural hematoma is its ability to obtain the proper view of the cavity, which enhances the ability to reach total clearance and ensures complete drainage of the hematoma. However, its limitations are drain collection with insufficient width and the risk of injury to the surface of the brain.

In this case, we selected neuroendoscopy because of the massive collection of fluid in the subdural space. In addition, few studies have reported its use in drainage of chronic subdural hematomas [2-5, 8]. To our knowledge, this is the second report of the use of neuroendoscopy to approach massive bilateral chronic subdural hematomas in children [2]. However, despite the presence of a bilateral chronic hematoma, non-accidental head trauma was ruled out after deep investigation to the best of our knowledge.

Not all chronic subdural hematomas in children are related to non-accidental head injuries, and physicians should be advised of this fact [9]. Several studies have considered retinal hemorrhage in combination with bilateral chronic subdural hematoma and encephalopathy as a high-prediction triad to diagnose non-accidental trauma. Currently, there is insufficient evidence of an accurate correlation among non-accidental head trauma and the presence of the triad [10-13]. In addition, our patient had a previously compromised, less consistent brain, and probably because of the low density, the brain was more prone to developing extra-axial hemorrhage after a minor head trauma, considering the novel knowledge of brain biomechanics [14]. Another differential diagnosis of chronic subdural hematoma in children should be considered, such as metabolic disease and of arachnoid cyst rupture [15].

According to the Pittsburgh Infant Brain Injury Score for non-accidental head trauma [16], which evaluated 1,040 infants to better define the probability of non-accidental head trauma, children should be scored for abnormalities on dermatologic examination (2 points), age >=3 months (1 point), head circumference >85th percentile (1 point), and serum hemoglobin <11.2 g/dL (1 point). In addition, if a child is suspected to have experienced non-accidental head trauma according to the brain image finding, such as the bilateral chronic subdural hematoma, a score>=2 increases the sensitivity to 93.3% and specificity to 53%. Our patient only had age as a positive factor and had a previous neurologic disease, which could cause bias.

Neuroendoscopy allows for better understanding of the pathophysiology of subdural hematoma formation. In our patient, we observed a laceration in the arachnoid membrane and a clot adhering to the brain surface, showing the origin of the injury. In addition, neuroendoscopy ensures complete drainage of the hematoma [2-5, 8].

## Conclusions

Neuroendoscopy of a massive chronic subdural hematoma is proven to be useful and safe, providing a reliable vision of the anatomy of the surface of the brain and allowing for treatment following a minimally invasive approach. Although bilateral chronic subdural hematomas have been associated with abusive head trauma, diagnosis of such conditions should be accurate to avoid misdiagnosis.

## References

[1] Miyake H, Kajimoto Y, Ohta T, Kuroiwa T (2002). Managing subdural fluid collection in infants. Childs Nerv Syst.

[2] Beez T, Schmitz AK, Steiger HJ (2018). Endoscopic lavage of extensive chronic subdural hematoma in an infant after abusive head trauma: adaptation of a technique from ventricular neuroendoscopy. Cureus.

[3] Majovsky M, Masopust V, Netuka D, Benes V (2016). Flexible endoscope-assisted evacuation of chronic subdural hematomas. Acta Neurochir (Wien).

[4] Masopust V, Netuka D, Hackel M (2003). Chronic subdural haematoma treatment with a rigid endoscope. Minim Invasive Neurosurg.

[5] Cai Q, Guo Q, Zhang F (2019). Evacuation of chronic and subacute subdural hematoma via transcranial neuroendoscopic approach. Neuropsychiatr Dis Treat.

[6] Furtado LM, da Costa Val Filho JA, Holliday JB, da Silva Costa J, de Matos MA, Nascimento VA, Ramos Cavalcanti T (2020). Endoscopic third ventriculostomy in patients with myelomeningocele after shunt failure. Childs Nerv Syst.

[7] Furtado LM, da Costa Val Filho JA, Giannetti AV (2020). Proposed radiological score for the evaluation of isolated fourth ventricle treated by endoscopic aqueductoplasty [IN PRESS]. Childs Nerv Syst.

[8] Hellwig D, Kuhn TJ, Bauer BL, List-Hellwig E (1996). Endoscopic treatment of septated chronic subdural hematoma. Surg Neurol.

[9] Fung EL, Sung RY, Nelson EA, Poon WS (2002). Unexplained subdural hematoma in young children: is it always child abuse?. Pediatr Int.

[10] Lynøe N, Elinder G, Hallberg B, Rosén M, Sundgren P, Eriksson A (2017). Insufficient evidence for 'shaken baby syndrome' - a systematic review. Acta Paediatr.

[11] Pierre-Kahn V, Roche O, Dureau P, Uteza Y, Renier D, Pierre-Kahn A, Dufier JL (2003). Ophthalmologic findings in suspected child abuse victims with subdural hematomas. Ophthalmology.

[12] Elinder G, Eriksson A, Hallberg B (2018). Traumatic shaking: The role of the triad in medical investigations of suspected traumatic shaking. Acta Paediatr.

[13] Kelly JP, Feldman K, Wright J, Ganti S, Metz JB, Weiss A (2020). Retinal and visual function in infants with non-accidental trauma and retinal hemorrhages. Doc Ophthalmol.

[14] van Zandwijk JP, Vester MEM, Bilo RA, van Rijn RR, Loeve AJ (2019). Modeling of inflicted head injury by shaking trauma in children: what can we learn?. Part II: A systematic review of mathematical and physical models. Forensic Sci Med Pathol.

[15] Furtado LM, Costa Val Filho JA, Ferreira RI, Mariano IG (2019). Intracranial arachnoid cyst rupture after mild TBI in children: have we underestimated this risk?. BMJ Case Rep.

[16] Berger RP, Fromkin J, Herman B (2016). Validation of the Pittsburgh Infant Brain Injury Score for abusive head trauma. Pediatrics.

